# Antioxidant Activity of *Graptopetalum paraguayense* E. Walther Leaf Extract Counteracts Oxidative Stress Induced by Ethanol and Carbon Tetrachloride Co-Induced Hepatotoxicity in Rats

**DOI:** 10.3390/antiox8080251

**Published:** 2019-07-28

**Authors:** Wen-Wan Chao, Shu-Ju Chen, Hui-Chen Peng, Jiunn-Wang Liao, Su-Tze Chou

**Affiliations:** 1 Department of Nutrition and Health Sciences, Kainan University, Taoyuan 33857, Taiwan; 2 Department of Food and Nutrition, Providence University, Taichung 43301, Taiwan; 3 Graduate Institute of Veterinary Pathobiology, National Chung Hsing University, Taichung 40227, Taiwan

**Keywords:** *Graptopetalum paraguayense* E. Walther, ethanol, carbon tetrachloride, antioxidant activity, hepatoprotective

## Abstract

(1) Background: *Graptopetalum paraguayense* E. Walther is a traditional Chinese herbal medicine. In our previous study, 50% ethanolic *G. paraguayense* extracts (GE50) demonstrated good antioxidant activity. (2) Methods: To investigate the hepatoprotective effects of GE50 on ethanol and carbon tetrachloride (CCl_4_) co-induced hepatic damage in rats, Sprague–Dawley rats were randomly divided into five groups (Control group; GE50 group, 0.25 g/100 g BW; EC group: Ethanol + CCl_4_, 1.25 mL 50% ethanol and 0.1 mL 20% CCl_4_/100 g BW; EC + GE50 group: Ethanol + CCl_4_ + GE50; EC + silymarin group: ethanol + CCl_4_ + silymarin, 20 mg/100 g BW) for six consecutive weeks. (3) Results: Compared with the control group, EC group significantly elevated the serum aspartate aminotransferase (AST), alanine aminitransferase (ALT), and lactate dehydrogenase (LDH). However, GE50 or silymarin treatment effectively reversed these changes. GE50 had a significant protective effect against ethanol + CCl_4_ induced lipid peroxidation and increased the levels of glutathione (GSH), vitamin C, E, total antioxidant status (TAS), and the activities of superoxide dismutase (SOD), glutathione peroxidase (GPx), catalase (CAT), and glutathione S-transferases (GST). Furthermore, in EC focal group, slight fat droplet infiltration was observed in the livers, while in the GE50 or silymarin treatment groups, decreased fat droplet infiltration. HPLC phytochemical profile of GE50 revealed the presence of gallic acid, flavone, genistin, daidzin, and quercetin. (4) Conclusions: The hepatoprotective activity of GE50 is proposed to occur through the synergic effects of its chemical component, namely, gallic acid, flavone, genistin, daidzin, and quercetin. Hence, *G. paraguayense* can be used as a complementary and alternative therapy in the prevention of alcohol + CCl_4_-induced liver injury.

## 1. Introduction

The liver, being a dynamic and vital organ, actively participates in multi-metabolic functions of foods, drugs, chemicals, biologicals, and xenobiotics, as well as detoxification of viral and bacterial products. These models, induced by toxins such as carbon tetrachloride (CCl_4_), dimethylnitrosamine (DMN), acetaminophen, or thioacetamide, can represent chronic or acute/fulminant hepatitis. Experimentally induced cirrhotic response in rat by CCl_4_ is shown to be similar to liver cirrhosis in the humans. Hepatotoxicity of CCl_4_ is largely due to its degraded metabolites trichloromethyl (CCl_3_) and trichloromethyl peroxyl (CCl_3_O_2_) formed by hepatic microsomal enzyme [1,2]. The hepatotoxicity of CCl_4_ is considered to be mediated by highly reactive trichloromethyl free radical (CCl_3_•) and/or peroxyl radical (CCl_3_OO•) activated forms of CCl_4_ formed by the action of the cytochrome P450 system, including CYP2B1, CYP2B2, CYP2E1, and CYP3A [3]. Liver injury induced by CCl_4_ has been widely used as an experimental model to screen medicine drugs [4,5,6].

Alcoholic liver disease (ALD), one of the most common causes of chronic liver disease in the world, is mainly caused by the excessive intake of alcohol. Its misuse represents a major risk factor for many organs, including the heart, brain, pancreas, and particularly, the liver. Oxidative stress associated with alcohol toxicity is mainly caused by reactive oxygen species (ROS) generated by the mitochondrial respiratory chain [7]. Oxidative stress is considered to be a key risk factor in the development of hepatic diseases. Ethanol is metabolized into acetaldehyde by alcohol metabolizing enzymes, including alcohol dehydrogenase (present in cytosol), CYP2E1 (present in microsomes), and catalase (CAT) (present in peroxisomes). The acetaldehyde is further oxidized into acetic acid by aldehyde dehydrogenase in the mitochondria [8]. Chronic alcohol consumption induces an increase in cellular nicotinamide adenine dinucleotide hydrate concentration and acetaldehyde dehydrogenase activity, which leads to severe free fatty acid overload, triglyceride accumulation, and subsequent steatosis in hepatic tissue [9]. Alcohol consumption is a common cause of death in adults, and CCl_4_ is a commonly used model for the hepatoprotective activity of drugs.

Plant phenols, such as flavonoids and anthocyanins, are widely distributed in the human diet through vegetables, fruits, cereals, beans, coffee, tea, natural herbs, and extracts, and they have been found to possess significant antioxidant activities. Silymarin is the extract of *Silybum marianum* and consists of seven flavonoglignans (silibinin, isosilibinin, silychristin, isosilychristin, and silydianin) and a flavonoid (taxifolin). *Silybum marianum* is one of the oldest and thoroughly researched plants in the treatment of liver diseases. Silymarin, found in milk thistle, also inhibits CYP2E1. Silymarin demonstrated potent antioxidative, anti-inflammatory, and immunomodulatory activities against liver diseases in various animal models [10,11]. Silymarin, a clinical antifibrotic agent, is widely accepted and used for treating liver diseases. 

*Graptopetalum paraguayense* E. Walther is a traditional Chinese herbal medicine belonging to the *Crassulaceae* family. In Taiwan, *G. paraguayense* is a medicinal plant that is regarded as a vegetable with health benefits. The general composition of *G. paraguayense* contains 95–96% water, 0.82% dietary fiber, 0.54% protein, 0.52% fat, and the total phenolic compounds and anthocyanins levels are in the range of 11—34 mg gallic acid equivalent/g and 0.03–1.29 μmol/g [12]. It has also demonstrated dose-dependent ACE inhibitory activity, and the kinetics of ACE inhibition reveal that the *G. paraguayense* extracts are mixed-type inhibitors [13]. Our previous study also demonstrated that the leaf extracts of *G. paraguayense* are safe and have a potential antioxidative activity [14,15,16]. The previous studies have shown that *G. paraguayense* plays neuroprotective effects of brain injury in ischemic rats [17] and reduces oxidative stress in hypercholesterolemia patients [18]. Furthermore, according to the Chinese prescription, *G. paraguayense* is able to alleviate hepatic disorders. In our previous studies, we have shown that *G. paraguayense* have hepatoprotective effects in human hepatoma cell line-HepG2 by induced G1 phase arrest and apoptosis [14]. In vivo, *G. paraguayense* can attenuate toxin-induced hepatic damage and fibrosis, while in vitro, it can inhibit HSC and Kupffer cell activation. Duh et al. suggested that *G. paraguayense* exerts hepatoprotection through antioxidative and anti-inflammatory properties and can protect against CCl_4_-induced oxidative stress and liver injury [19]. The treatment of a rat model with diethylnitrosamine (DEN)-induced liver cancer with *G. paraguayense* extracts decreased hepatic collagen contents and inhibited tumor growth [20]. The abovementioned suggest that *G. paraguayense* could be considered as a complementary therapy agent for liver disease [21]. In this study, we investigated the protective effects of *G. paraguayense* extracted with 50% ethanol (GE50) against ethanol + CCl_4_ (EC)-induced hepatic damage in rats. Silymarin, an antioxidant flavonoid, was used as the control preventive agent in our experiment.

## 2. Materials and Methods

### 2.1. Preparation of GE50 Extract of G. Paraguayense

The 50% ethanolic extract of *G. paraguayense* E. Walther (GE50) was prepared according to the previously described procedures [12]. The *G. paraguayense* E. Walther was grown in a pot, and the leaves were cleaned, washed, cut into small pieces, and then freeze-dried by a vacuum freeze-dryer. Each 20 g of freeze-dried leaves was extracted with 700 mL of 50% ethanol at 85 °C for 3 h. The decoction was filtered and dried using a vacuum freeze-dryer. The GE50 extracts were sealed in plastic bottles and stored at −70 °C until use.

### 2.2. Animal Treatment

Fifty male weanling Sprague–Dawley (SD) rats were obtained and fed commercial chow diets (Fwusow Industry Co., LTD, Taiwan). They were randomly divided into five groups, each containing ten animals. The control group was gavaged with 1.25 mL of normal saline daily for six weeks. The GE50 group was gavaged with GE50, dissolved in 1.25 mL normal saline, at a dose of 0.25 g/100 g BW for six weeks. The ethanol + CCl_4_ (EC) group was gavaged with 50% ethanol 1.25 mL 50% ethanol/100 g BW (equal to 0.5 g ethanol/100 g BW) and 0.1 mL of 20% CCl_4_ in olive oil twice a week and administered 1.25 mL of normal saline daily for six weeks. The ethanol + CCl_4_ + GE50 (EC + GE50) group and ethanol + CCl_4_ + silymarin (EC + silymarin) group were gavaged with GE50 (0.25 g/100 g BW) and silymarin (20 mg/100 g BW), respectively, daily for six weeks and received ethanol/CCl_4_ in the same manner as EC group. The control and GE50 groups, which are not administered CCl_4_, received 0.1 mL of olive oil/100 g BW at the same time points. The animals were kept under standard laboratory conditions of light/dark cycle, a temperature of 22 ± 2 °C and humidity of 50 ± 10%. This animal research and all the procedures were reviewed and approved by the Animal Research Ethics Committee at Providence University, Taichung, Taiwan (Approval No: 20071210-A05).

### 2.3. Serum and Liver Tissue Preparation

After six weeks of feeding, the blood was collected. The serum was analyzed for aspartate aminotransferase (AST), alanine aminotransferase (ALT), lactate dehydrogenase (LDH), and total antioxidant status (TAS). The livers were homogenized in an ice-cold phosphate buffer (0.05 M, pH 7.4) using a Potter-Elvehjem-type homogenizer with a Teflon pestle. One portion of this tissue homogenate (0.3 g/mL) was used for assaying the levels of malondialdehyde (MDA), vitamin C, vitamin E, and reduced glutathione (GSH). After centrifuged at 12,000× *g* and 4 °C for 10 min. The resulting supernatant was used to determine the activities of superoxide dismutase (SOD), glutathione peroxidase (GPx), CAT, and glutathione S-transferase (GST). 

### 2.4. Determination of AST, ALT, LDH, and TAS Serum Levels in Rats

The levels of AST and ALT in the serum samples were determined by enzymatic methods using an automatic analyzer at a commercial analytical service center (Lian-Ming Co., Taiwan, ROC). The levels of LDH and TAS were determined using commercial kits from Randox Laboratories Ltd. (Antrim, UK).

### 2.5. Measurement of *MDA* and GSH Levels, and GPx, SOD, and CAT, and GST Activities 

To measure activities in the liver, MDA, GSH, GPx, SOD, and CAT were performed in accordance with our previously reported procedures. Tissue MDA levels were used to spectrophotometrically estimate thiobarbituric acid-reactive substances (TBARS) at 535 nm. GSH contents were measured by HPLC. SOD activity was determined spectrophotometrically at 325 nm. One unit of SOD activity was defined as half the rate of reduction of pyrogallol autoxidation over a 1-min period at 15-s intervals. GPx activity was determined by an enzyme coupled method with glutathione reductase (GR) using cumene hydroperoxide as the substrate at 30 °C. The rate of decrease in the NADPH concentration was observed at 340 nm over a 3-min period at 30-s intervals. One unit of GPx activity was defined as the amount of enzyme that catalyzed the oxidation of 1 μmol of NADPH/min/mL. CAT activity was determined using H_2_O_2_ as the substrate. The rate of H_2_O_2_ dismutated to H_2_O and O_2_ was proportional to the CAT activity. The decrease in the amount of H_2_O_2_ was observed at 240 nm over a 1-min period at 15-s intervals. One unit of CAT activity was defined as 1 mmole H_2_O_2_ remaining per minute [22]. GST activity was determined at 340 nm by an enzyme-coupled method with glutathione-1-chloro-2,4-dinitrobenzene (CDNB) as the substrate at 25 °C [23]. One unit of GST activity was defined as the amount of enzyme that catalyzed the formation of glutathione-CDNB/min/ml. The protein content of the tissue cytosols was determined based on the Biuret reaction using a BCA kit. The specific activity of the enzyme was expressed as unit/mg protein.

### 2.6. Measurement of Antioxidants

The liver vitamin C content was stabilized by MPA and cysteine solution and determined by HPLC [24]. The vitamin E standard and the tissue cytosols were diluted in methanol solution containing 0.25% BHT and 0.2% ascorbate before HPLC analysis. The tissue vitamin E and GSH contents were measured by HPLC [25,26]. The tissue GSH was reduced by dithiothrietol and the monobromobimane derivative was produced before the HPLC analysis. As the eluent, 30 mM TBA in methanol solution was used at a flow rate of 1 mL/min.

### 2.7. Histopathology

The liver tissue of rats was immobilized in 10% formalin solution for 24 h. Then, the liver tissue was dehydrated, made transparent, waxed, embedded, and sectioned. Liver tissues were trimmed to 2-mm thickness. Then, the liver tissue was stained with hematoxylin and eosin (H&E). 

### 2.8. Characterization of Phenolic Compounds in GE50 Extracts

Determine the polyphenolic compounds in GE50 extracts, HPLC analysis was performed in accordance with our previously described method with modifications [16,27]. GE50 was dissolved in 50% ethanol, filtered, and analyzed by HPLC. Peak areas and concentrations were determined. The identification of each compound was based on a combination of retention time and spectral matching by comparison with those of known standards.

### 2.9. Statistical Analysis 

Data are expressed as mean ± standard deviation (SD). Statistical analyses were conducted using SPSS (v.16.0; SPSS, USA). One-way ANOVA and Scheffe’s method were used to analyze the differences between the means. Differences with *p* < 0.05 were considered statistically significant.

## 3. Results

### 3.1. Effects of GE50 on Body Weight Gain, Feed Efficiency, and Liver Index in Ethanol + CCl_4_ -Treated Group 

Table 1 shows the daily body weight gain, feed efficiency, and liver index of the rats in each group. The effects of GE50 on the body weight were evaluated. Silymarin, a polyphenolic flavonoid, was used in this study as a reference drug. Significant weight loss was observed in ethanol + CCl_4_ (EC)-treated group. The change in body weight was highest in the EC-treated group (3.56 ± 0.66 g/day/rat) compared with the control (5.58 ± 0.66 g/day/rat), followed by the GE50 (5.92 ± 0.81 g/day/rat), EC + GE50 (4.68 ± 0.59 g/day/rat), and EC + silymarin (3.56 ± 0.74 g/day/rat) groups. The change in feed efficiency was the highest in EC-treated group compared with the other groups. The relative liver weight in the EC-treated group was the highest compared with the other groups. The administration of GE50 or silymarin over six weeks significantly reversed the ethanol + CCl_4_ effects, inducing body weight gain and improving feed efficiency.

### 3.2. Effects of GE50 on Ethanol + CCl_4_ -Induced Hepatotoxicity

To evaluate possible hepatocellular damage caused by ethanol and CCl_4_, the activities of AST and ALT were assessed. Ethanol and CCl_4_ co-treatment significantly increased the activity of these enzymes in plasma, indicating intense hepatic damage. As shown in Table 2, the serum AST, ALT, LDH, and TAS levels were measured. A significantly higher serum levels of AST, ALT, and LDH were observed in the EC group than in the control group (AST: 184.40 ± 25.5 vs. 103.98 ± 14.0 U/L; ALT: 66.89 ± 4.9 vs. 44.10 ± 5.4 U/L; LDH: 563.04 ± 103.7 vs. 273.82 ± 94.9 U/L). However, EC + GE50 treatment efficiently decreased the AST level to 110.95 ± 14.0 U/L, ALT level to 50.01 ± 4.2, and LDH level to 403.20 ± 79.4 U/L. Similar results were obtained when the rats were treated with silymarin, which is a known hepatoprotective chemical.

The serum TAS level in rats showed a significantly decreased in the EC group compared to the control group (0.15 ± 0.1 vs. 0.39 ± 0.1 nmole/L). The EC + GE50 treatment reversed the TAS level to 0.28 ± 0.1 nmole/L. GE50 treatment alone did not affect the serum AST and ALT levels.

### 3.3. Histological Analyses

The hepatoprotective effects were confirmed by histological examinations. Hepatic steatosis represents an excess accumulation of fat in hepatocytes. To assess the degree of fatty liver, we examined the accumulation of hepatic triglyceride by H&E staining of the rat liver. Ethanol administration caused degenerative morphological changes, which were exhibited by fat droplets in the liver sections.

**Figure 1:**
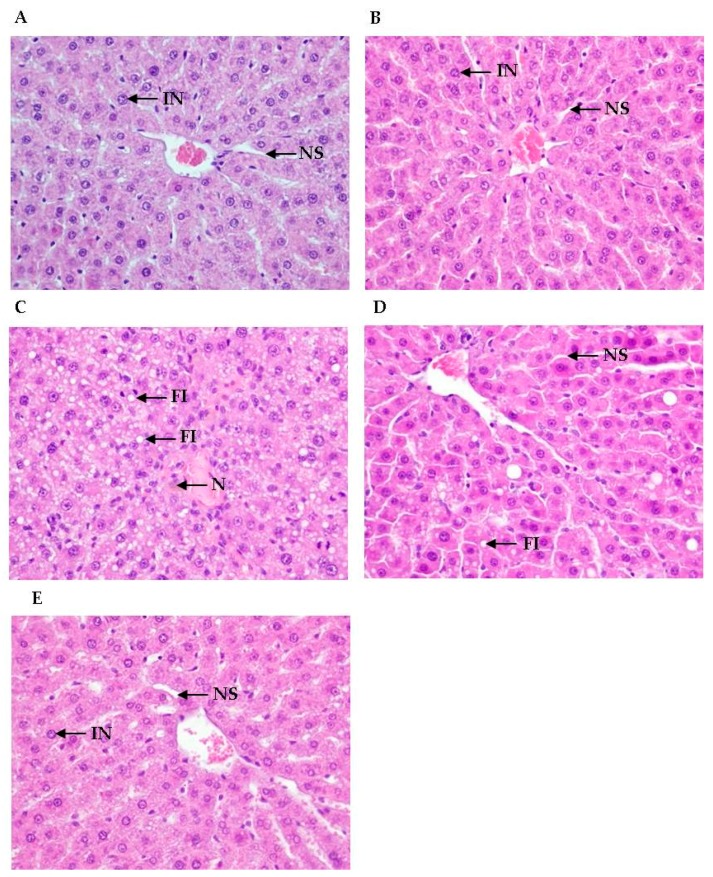
Effects of oral administration of GE50 over six consecutive weeks on histopathological changes in the livers of rats treated with ethanol + CCl_4_. Hematoxylin/Eosin staining (H&E), 400×. (**A**) Liver section from the control group demonstrating normal hepatic architecture with intact nucleus (IN) and normal sinusoids (NS); (**B**) GE50; (**C**) Section of ethanol + CCl_4_ (EC)-induced damaged liver showing highly deformed hepatic architecture with fatty lesion due to fatty infiltration (FI) and necrosis (N); **(D**) EC+ GE50; **(E**) Liver section from EC + silymarin-treated group showing improved hepatocellular architecture with IN and NS.

The results of H&E staining indicated that ethanol and CCl_4_ co-administration presented extensive changes in the liver morphology, including marked enlarged areas of hepatocellular degeneration and infiltration inflammatory cells. No histological abnormality was observed in the control group. As illustrated in Figure 1, ethanol + CCl_4_ (EC)-induced injury included increased vacuole formation, neutrophil infiltration, and necrosis. Liver section from the EC-treated group showed highly deformed liver architecture with fatty lesion due to intensive fatty infiltration (FI) and signs of necrosis (N). EC + GE50 group and EC + silymarin group demonstrated improved hepatocellular architecture with intact nucleus (IN) and normal sinusoids (NS). 

### 3.4. Effects of GE50 on Hepatic MDA, Vitamin C, Vitamin E, and GSH Levels in Ethanol + CCl_4_-Treated Group 

**Figure 2:**
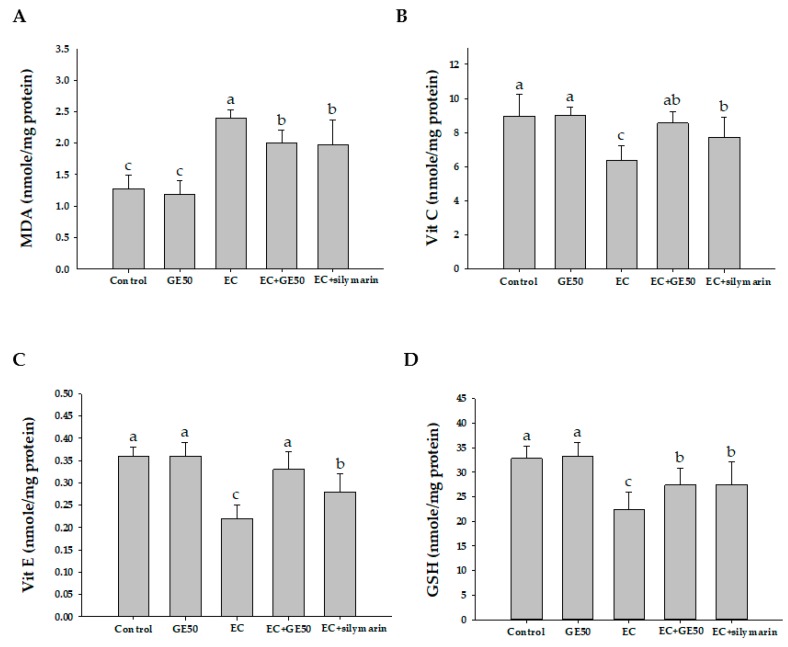
Effects of oral administration of GE50 over six consecutive weeks on malondialdehyde (MDA) (**A**), vitamin C (**B**), vitamin E (**C**), glutathione (GSH) (**D**) levels in the livers of rats treated with ethanol + CCl_4_. The data are presented as mean ± S.D. of 10 rats. One-way ANOVA and Scheffe’s method were used to analyze the differences between the means. ^a–^^c^ Mean values with different letters in the same row are significantly different (*p* < 0.05) according to Duncan’s multiple-range test. Control group; GE50 group; EC group: Ethanol + CCl_4_; EC + GE50 group: Ethanol + CCl_4_ + GE50; EC + silymarin group: ethanol + CCl_4_ + silymarin.

In our study, the hepatic MDA levels were significantly elevated in the ethanol + CCl_4_ (EC) group (2.40 ± 0.13 nmol/mg protein) compared with that in the control (1.28 ± 0.21 nmol/mg protein) and GE50 (1.19 ± 0.21 nmol/mg protein) groups (*p* < 0.05). In contrast, in the EC + GE50 group and EC + silymarin group, hepatic MDA levels were markedly decreased compared with the EC group (Figure 2). This observation indicated that the plant extracts may provide phytochemicals that inhibit lipid peroxidation in the rat liver.

Vitamin C level was significantly decreased in the EC group (6.37 ± 0.87 nmol/mg protein) compared with the control group (8.97 ± 1.26 nmol/mg protein). Vitamin E level was also significantly decreased in the EC group (0.22 ± 0.03 nmol/mg protein) compared with the control group (0.36 ± 0.02 nmol/mg protein). GSH level was significantly decreased in the EC group (22.30 ± 3.62 nmol/mg protein) compared with the control group (32.78 ± 2.55 nmol/mg protein). However, EC + GE50 treatment reversed the vitamin C level to 8.55 ± 0.68 nmol/mg protein, vitamin E level to 0.33 ± 0.04 nmol nmol/mg protein, and GSH level to 27.41 ± 3.45 nmol/mg protein. The decline of GSH level in the EC group might be due to its excessively generated quantity of free radicals leading to hepatic injury.

### 3.5. Effects of GE50 on Antioxidant Enzymatic Activities in Ethanol + CCl_4_-Treated Group

**Figure 3:**
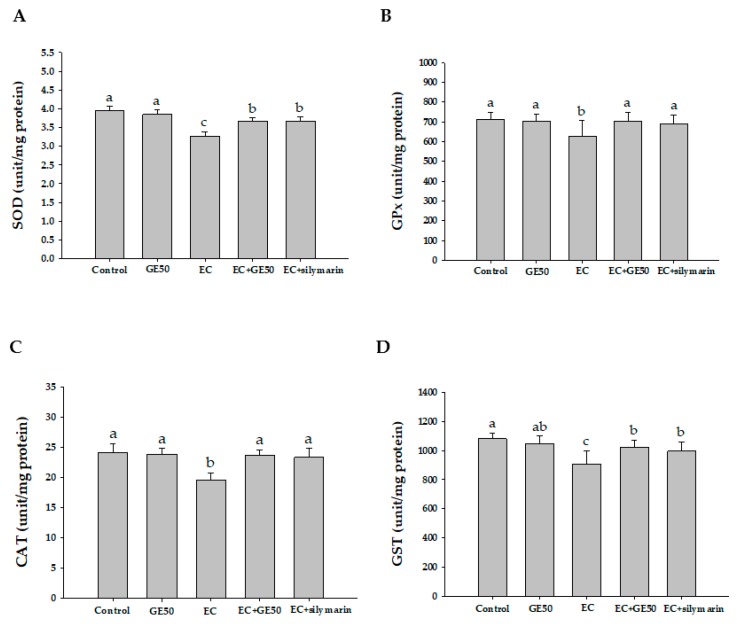
Effects of oral administration of GE50 over six consecutive weeks on superoxide dismutase (SOD) (**A**), glutathione peroxidase (GPx), (**B**), catalase (CAT), (**C**), and glutathione S-transferases (GST) (**D**) activities in the livers of rats treated with ethanol + CCl_4_. The data are presented as mean ± S.D. of 10 rats. One-way ANOVA and Scheffe’s method were used to analyze the differences between the means. ^a–c^ Mean values with different letters in the same row are significantly different (*p* < 0.05) according to Duncan’s multiple-range test. Control group; GE50 group; EC group: Ethanol + CCl_4_; EC + GE50 group: Ethanol + CCl_4_ + GE50; EC + silymarin group: ethanol + CCl_4_ + silymarin.

The activities of SOD, GPx, CAT, and GST were measured to evaluate the antioxidant effects of GE50 (Figure 3). The SOD activity significantly decreased in the ethanol + CCl_4_ (EC) group (3.27 ± 0.11 unit/mg protein) compared with the control (3.95 ± 0.11 unit/mg protein) and GE50 (3.91 ± 0.19 unit/mg protein) groups (*p* < 0.05). The GPx activity significantly decreased in the EC group (627.25 ± 79.43 unit/mg protein) compared with the control (711.73 ± 37.97 unit/mg protein) and GE50 (702.96 ± 37.71 unit/mg protein) groups. The CAT activity significantly decreased in the EC group (19.61 ± 1.11 unit/mg protein) compared with the control (24.11 ± 1.44 unit/mg protein) and GE50 (23.87 ± 0.91 unit/mg protein) groups. The GST activity significantly decreased in the EC group (908.03 ± 88.92 unit/mg protein) compared with the control (1079.68 ± 41.13 unit/mg protein) and GE50 (1045.72 ± 52.27 unit/mg protein) groups (*p* < 0.05). GE50 treatment successfully recovered these enzymes levels to almost normal levels. The effect of GE50 was similar to silymarin, which has been previously shown to have a significant protective effect in rats. 

### 3.6. Polyphenolic Profile in GE50

**Figure 4:**
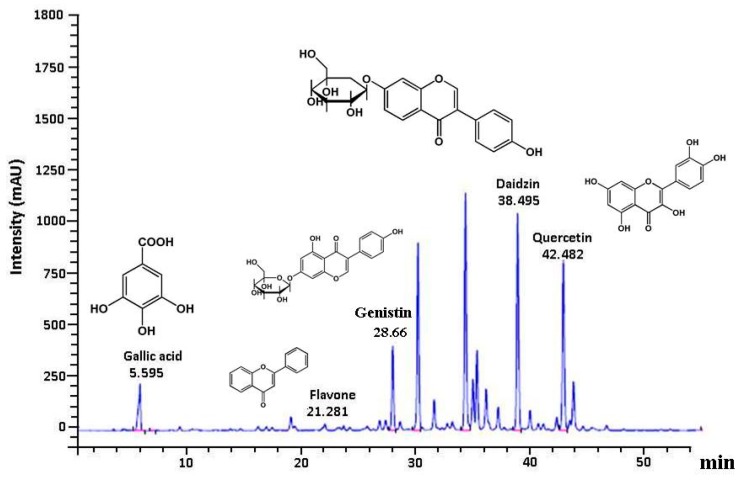
HPLC phytochemical profile of GE50 extract detected at 270 nm. GE50 chemical profile was identified by relative retention times using authentic standards. Key to peak identities: Gallic acid, flavone, genistin, daidzin, and quercetin.

The HPLC chromatogram showed that gallic acid, quercetin, genistin, and daidzin were the major components among organic molecules found in GE50, which had maximum absorbance at 270 nm. *G. paraguayense* E. Walther mainly contained flavonoids that were identified in our study (Figure 4). It has also been reported that *G. paraguayense* E. Walther contains various antioxidants, such as gallic acid, quercetin, genistin, and daidzin [16,17]. The protective effects of GE50 may be attributed to the presence of polyphenolic compounds such as gallic acid, flavone, genistin, daidzin, and quercetin. The pharmacological fundamental constituents of the plant are flavonoids.

## 4. Discussion

CCl_4_ is a typical hepatotoxic substance, and its mechanism of action is complex. CCl_4_-mediated hepatotoxicity was chosen as the experimental model. Due to ethanol and CCl_4_ toxicity, relative liver weight in the ethanol + CCl_4_ (EC)-treated group was higher than that in the control group (Table 1). Liver weight is known to increase due to hepatic damage inflicted by trichloromethyl radical, as well as consequent liver fibrosis and hypertrophy. Changes in body and liver weight after ethanol and CCl_4_ intoxication provides direct evidence of the overall hepatic damage [28,29]. The liver, the largest and the most metabolically complex organ in the body, is involved in the storage and biosynthesis metabolism. It is also responsible for detoxification and metabolic homeostasis. Ethanol and CCl_4_ resulted in loss of body weight, which is considered a putative indicator of health. We demonstrated that GE50 markedly ameliorated ethanol + CCl_4_ (EC)-induced chronic hepatitis in test rats, accompanied by reduced relative liver weight. Similar results were obtained when the rats were treated with silymarin, a commercial hepatoprotective agent. Silymarin was used as the positive control in this study.

AST and ALT are aminotransferases linked with liver parenchymal cells. If the hepatocellular plasma membrane is damaged, these will leak out from the cytosol into the bloodstream. Serum AST and ALT levels markedly increased in the ethanol + CCl_4_ (EC)-treated group, indicating altered permeability of membranes and hepatotoxicity. The results revealed that the serum AST and ALT levels significantly decreased after treatment with GE50. These results demonstrate the hepatoprotective effect of GE50 against ethanol + CCl_4_-induced liver injury in rats. Damage to the liver cells results in elevations of the both ALT and AST, which have been widely accepted as major biomarkers to assess the hepatic injury [30].

Alcoholic hepatitis should at least include inflammation, steatosis, fibrosis, and cell damage. The activity of alcohol dehydrogenase and aldehyde dehydrogenase causes a reduction in NAD^+^/NADH ratio, which is the process that causes a reduced mitochondrial capacity to metabolize lipids [11,31]. Otherwise, CCl_4_-induced liver injury is characterized by oxidative stress and activated hepatic macrophage, leading to hepatocyte damage and death [32]. Chronic alcohol consumption increases cellular nicotinamide adenine dinucleotide hydrate concentration and acetaldehyde dehydrogenase activity, which leads to severe free fatty acid overload, triglyceride accumulation, and subsequent hepatic steatosis [9]. Liver injury can lead to the transfer of fatty acids to the liver, resulting in an increase in the TG content in the liver. These results demonstrate that GE50 treatment significantly alleviated the degree of liver injury.

Lipid peroxidation is one of the major characteristics of CCl_4_-induced hepatotoxicity [33]. MDA, the end product of lipid peroxidation, is widely used as a marker of lipid peroxidation injury. Antioxidants, such as N-acetyl-cysteine, α-tocopherol, phenols, selenium, and vitamin C and E, have been proposed and used as therapeutic agents in liver damage [34]. Vitamin E is believed to be the most important lipophilic antioxidant in biological tissues. Cheeseman et al. demonstrated that an increased vitamin E level in liver protects from acute CCl_4_-induced damage by preventing lipid peroxidation [35].

Astrocytes contain one of the highest cytosolic concentrations of GSH amongst mammalian cells. GSH is the major non-protein thiol that plays a vital role in maintaining the antioxidant defense mechanism in the body. GSH levels in the liver dropped in CCl_4_-treated mice. The depletion of GSH may also be a consequence of liver damage. Ethanol inhibits the synthesis of reduced GSH. Moreover, acetaldehyde promotes GSH depletion, free radical-mediated toxicity, and lipid peroxidation [36]. The hepatoprotective effects of some compounds, such as silymarin, may be attributable to its ability to increase cellular GSH [37]. The increase in hepatic GSH by GE50 may be one of the ways in which *G. paraguayense* protects the liver against ethanol and CCl_4_ co-induced hepatotoxicity in rats.

CCl_4_ initiates lipid peroxidation, as well as reduces tissue GPx, GR, CAT, and SOD activities. In experimental animals, the induction of an SOD–CAT-insensitive free radical species by diet and alcohol facilitates liver damage [38]. The GST family represents one of the main detoxification systems in the hepatocytes. GST regulates apoptosis by influencing the lipid peroxidation pathway [39]. GPx and GSH are well-known reductants that metabolize toxic chemicals, drugs, and xenobiotics. In general, the liver can combat the free radical damage by biotransformation of these toxic agents in less reactive compounds through cytochrome P-450 and GPx [40]. CAT plays a role in the metabolism of ethanol. In addition to alcohol dehydrogenase and CYP2E1, CAT is involved in the metabolism of ethanol in the body. 

The treatment of SD rats with alcohol and CCl_4_ caused the development of significant hepatocellular damage, as was evident from a marked increase in the serum activities of AST, ALT, and LDH compared with the control rats (Table 2). We observed a large number of inflammatory cell infiltration in the ethanol + CCl_4_ (EC) co-treatment group (Figure 1). Rats treated with the GE50 had lower concentration of MDA in the liver cells. Alcohol + CCl_4_ (EC) co-treatment also caused a considerable increase (*p* < 0.05) in hepatic MDA formation (Figure 2) and simultaneously induced a marked depletion (*p* < 0.05) in vitamin C, vitamin E, GSH, and SOD, GPx, CAT, and GST levels in the liver (Figure 3) compared with the control rats. Our studies showed a decrease in food intake and increase in oxidative stress in rats co-treated ethanol and CCl_4_. Our results demonstrated that GE50 significantly enhanced the GSH, SOD, CAT, and GR levels, and may contribute to the important mechanisms underlying the liver preventive effects. 

Plant flavonoid compounds are a gifted class of so-called “nutraceuticals” that include the ability to protect hepatic damage [41,42,43]. Phytochemicals are naturally occurring chemicals in plant that promote the prevention and treatment of various diseases. Plants are a good source of useful hepatoprotective agents that can modulate the activities of free radicals [41]. A significant decrease in lipid peroxides in liver tissues following co-treatment with CCl_4_ and antioxidants indicated the protective effects of polysaccharide from *Angelica* and *Astragalus* [44] and *Fagonia schweinfurthii* [45] through the scavenging of free radicals produced by CCl_4_. Several reports have shown that *Antrodia camphorate*, also known as *Antrodia cinnamomea* (*Niuchangchih*), is a precious fungus grown in Taiwan. It has been reported to prevent ethanol-, CCl_4_-, and cytokine-induced liver injury, ameliorate fatty liver and liver fibrosis, and inhibit hepatoma cells [46,47,48]. In our study, GE50 and *Niuchangchih* were identical to the positive drug Silymarin.

The structural characteristics of plant polyphenols provide them with strong antioxidant and free radical scavenging abilities. Hepatoprotection using edible phytochemicals is a novel strategy for the treatment of various hepatic dysfunctions. Gallic acid (3,4,5-trihydroxybenzoic acid), a phenolic acid with strong antioxidant effect, is abundant in tea leaves, as well as white, red, and black mulberry, blackberry, raspberry, strawberry, dragon fruit, guava, mangosteen, papaya, and other plants. Gallic acid downregulated CYP2E1 expression in liver tissues while increasing SOD activities. These results support its ability to regulate the antioxidant enzymes activities and inhibit lipid peroxidation. Many studies have demonstrated its hepatoprotective effects [49,50,51]. 

Isoflavones are dietary phytoestrogens occurring naturally in legumes such as soybeans. Two major isoflavones found in soybean are daidzin and genistin, respectively. In soy and soy products, 95–99% of genistein exists in the conjugated form genistin (glycoside). Many studies demonstrated that daidzein and genistein alleviate hepatic damage [52,53]. 

Quercetin (3,5,7,30,40-pentahydroxyflavone) is a major flavonoid found in fruits, vegetables, and red wine and displays several healthy properties, including antioxidative, anti-inflammatory, anti-apoptotic, and hepatoprotective activities against various hepatic ailments. Several studies examined the protective effects of quercetin on chronic ethanol-induced liver injury [43,54]. Quercetin ameliorated lipid metabolism and ethanol-induced liver damage by inducing antioxidant enzymes, increasing GSH levels, and reducing CYP2E1 activity [55]. Quercetin, a well-known flavonoid, ameliorates CCl_4_ and ethanol-induced liver injury in vivo by alleviating oxidative stress [56,57,58]. The antioxidant and antifibrotic effect of GE50 may be due to presence of quercetin. In our previous study, we also demonstrated that GE50 contains high levels of quercetin. The major components isolated were quercetin 3-O-[6′’-(3-hydroxyl-3-methylglutaroyl)]-β-d- glucopyranoside and quercetin 3-O-[6´´-(3-hydroxyl-3-methylglutaroyl)-2´´-acetyl]- β-d-glucopyranoside [16]. Quercetin also improves the antioxidant defense mechanism by increasing the levels of enzymatic and nonenzymatic antioxidants in cells, thus reducing oxidative stress.

It is, therefore, reasonable to assume that the hepatoprotective activities of GE50 is attributed to its gallic acid, flavone, genistin, daidzin, and quercetin components that most possibly act synergistically. *G. paraguayense* is an edible vegetable that has also been used in traditional Taiwanese folk medicine for protection against liver injury.

## 5. Conclusions

In conclusion, the results of our study indicate that GE50 enhances hepatic antioxidant enzyme activities, as well as inhibits lipid peroxidation in ethanol and CCl_4_ (EC) co-induced liver injury, and its effect is similar to that of silymarin. The hepatoprotective activity of GE50 is proposed to occur through the synergic effects of its chemical component, namely gallic acid, flavone, genistin, daidzin, and quercetin. These results confirm that the in vivo hepatoprotective activity of GE50 may be associated with the phenolic phytochemicals present in the extract, which are known for their antioxidant potential. GE50 can be used as a functional food or even a pharmacological agent for the prevention of liver diseases.

## Figures and Tables

**Table 1:** Effects of oral administration of 50% ethanolic *Graptopetalum paraguayense* E. Walther (GE50) over six consecutive weeks on body weight gain, feed efficiency, and relative liver weight in rats treated with ethanol + carbon tetrachloride (CCl_4_).

Groups	Daily Body Weight Gain (g/Day/Rat)	Feed Efficiency (g Gain/g Feed)	Relative Liver Weight (g/100 g Body Weight)
Control	5.58 ± 0.66 ^a^	0.21 ± 0.02 ^a^	2.96 ± 0.14 ^c^
GE50	5.92 ± 0.81 ^a^	0.21 ± 0.02 ^ab^	3.05 ± 0.19 ^bc^
Ethanol + CCl_4_ (EC)	3.56 ± 0.66 ^c^	0.15 ± 0.01 ^c^	3.30 ± 0.11 ^a^
EC + GE50	4.68 ± 0.59 ^b^	0.20 ± 0.01 ^b^	3.10 ± 0.12 ^b^
EC + silymarin	4.32 ± 0.74 ^b^	0.20 ± 0.02 ^ab^	3.17 ± 0.11 ^b^

The data are presented as mean ± S.D. of 10 rats. One-way ANOVA and Scheffe’s method were used to analyze the differences between the means. ^a–^^c^ Mean values with different letters in the same row are significantly different (*p* < 0.05) according to Duncan’s multiple-range test. Control group; GE50 group, 0.25 g/100 g BW; EC group: Ethanol + CCl_4_, 1.25 mL 50% ethanol and 0.1 mL 20% CCl_4_/100 g BW; EC + GE50 group: Ethanol + CCl_4_ + GE50; EC + silymarin group: ethanol + CCl_4_ + silymarin, 20 mg/100 g BW.

**Table 2:** Effects of oral administration of GE50 over six consecutive weeks on serum aspartate aminotransferase (AST), alanine aminotransferase (ALT), and lactate dehydrogenase (LDH) levels, and total antioxidant status (TAS) in rats treated with ethanol + CCl_4_.

Groups	AST (U/L)	ALT (U/L)	LDH (U/L)	TAS (nmole/L)
Control	103.98 ± 14.0 ^c^	44.10 ± 5.4 ^c^	273.82 ± 94.9 ^c^	0.39 ± 0.1 ^a^
GE50	97.25 ± 19.8 ^c^	44.28 ± 3.8 ^c^	218.39 ± 35.2 ^c^	0.37 ± 0.1 ^a^
Ethanol + CCl_4_ (EC)	184.40 ± 25.5 ^a^	66.89 ± 4.9 ^a^	563.04 ± 103.7 ^a^	0.15 ± 0.1 ^c^
EC + GE50	110.95 ± 14.0 ^bc^	50.01 ± 4.2 ^b^	403.20 ± 79.4 ^b^	0.28 ± 0.1 ^b^
EC + silymarin	122.28 ± 24.2 ^b^	42.05 ± 6.6 ^c^	347.13 ± 43.9 ^b^	0.26 ± 0.1 ^b^

The data are presented as mean ± S.D. of 10 rats. One-way ANOVA and Scheffe’s method were used to analyze the differences between the means. ^a–^^c^ Mean values with different letters in the same row are significantly different (*p* < 0.05) according to Duncan’s multiple-range test. Control group; GE50 group; EC group: Ethanol + CCl_4_; EC + GE50 group: Ethanol + CCl_4_ + GE50; EC + silymarin group: ethanol + CCl_4_ + silymarin.
